# Early Health Economic Modelling – Optimizing Development for Medical Device Developers? Comment on "Problems and Promises of Health Technologies: The Role of Early Health Economic Modeling"

**DOI:** 10.15171/ijhpm.2019.136

**Published:** 2019-12-22

**Authors:** Conor Teljeur, Máirín Ryan

**Affiliations:** Health Information and Quality Authority, Dublin, Ireland.

**Keywords:** Health Technology Assessment, Economic Modelling, Medical Devices, Reimbursement

## Abstract

This commentary considers the positive and negative consequences of early economic modelling and explores potential future directions. Early economic modelling offers device manufacturers an opportunity to assess the potential value of an innovation at an early stage of development. Early modelling can direct resources into potentially viable technologies and reduce investment in technologies with limited prospect of value. However, it is unclear whether early modelling is sufficiently specific to identify innovations with low value. It may be that early modelling is more useful for directing data gathering to reduce decision uncertainty. Early modelling is of primary benefit to the manufacturer and may have both positive and negative consequences for reimbursement processes that should be considered.

## Introduction

Grutters and colleagues described the experience of carrying out early-stage economic models to inform product development.^1^ They reviewed 32 evaluations of 30 non-drug innovations that were used to support further development, research and implementation decisions. It is unclear to what extent the findings of the study can be generalised, and perhaps it should be viewed as a pilot study that highlights the potential of early economic modelling.

The subtle difference between a conventional and early economic evaluation is a combination of timing and whether it supports a decision by the manufacturer or the payer. In the context of pharmaceuticals, economic evaluation and an associated health technology assessment (HTA) is generally developed by the manufacturer as part of the process of seeking reimbursement. For devices, on the other hand, there are few established processes for reimbursement and a submission with economic modelling is not required as standard. Therefore device manufacturers do not routinely need to develop economic models for their products.

If one considers where economic modelling currently sits in product lifecycle, it commonly occurs at a late stage and may be viewed by the manufacturer as an impediment delaying reimbursement. By occurring earlier in the product lifecycle, economic evaluation is viewed as a positive contributor to product development rather than a barrier.^2^ The focus here is on economic evaluation, which forms only part of the information collected for a full HTA process. Rather than being limited to the outcome of an economic evaluation, reimbursement by payers is often contingent on a full HTA. This commentary considers the potential positive and negative consequences of early economic modelling for devices, and explores possible future directions.

## Potential Benefits

### Efficient Investment

Early economic modelling clearly has the potential to reduce investment in innovations that may be of limited clinical value or may be unlikely to be reimbursed on the grounds of cost-effectiveness. By directing investment and development efforts, early economic modelling may reduce costs by ceasing development of a product at an earlier stage, and this is clearly the aim. This is particularly true for small to medium enterprises, where a substantial investment in even one or a small number of technologies that ultimately prove to be non-viable could be the death knell for the company.

### Directing Data Collection

Early economic modelling can clearly serve an important role in directing data collection efforts. Evidence synthesis is costly and time consuming, and many technology developers may lack the resources and expertise to gather the types of data required for economic modelling. Early economic modelling can, for example, use value of information analyses to ensure that there is a focus on parameters that can reduce decision uncertainty and at the same time avoid pursuing information that may be costly to collect and is unlikely to have a major impact on decision-making.^2^

### Supporting Health Technology Assessment 

There is still substantial regional variation in how decisions are made regarding reimbursement of medical devices and non-pharmacological technologies. While the landscape is changing, it is still not at the stage where devices commonly go through a HTA appraisal process as is often the case for drugs. In the event that a formal HTA process is used, particularly an appraisal process analogous to that for pharmaceuticals, the use of early economic modelling can create efficiencies for the subsequent reimbursement application process if it reflects the standards for economic evaluation in HTA.

## Potential Disadvantages

### Focus on Cost-Effectiveness

The approach to early economic modelling as described by Grutters et al is first and foremost to benefit the manufacturer. The concept of value for money depends on the perspective, and in this case the manufacturer is seeking information that will ensure maximum return within the constraints of what the payer deems acceptable and affordable. By focusing on cost-effectiveness from an early stage, product development may become overly focused on cost-effectiveness and lead to the clustering of incremental cost-effectiveness ratios around the willingness-to-pay threshold. From a payer perspective, the willingness to pay threshold should not be a target, and early economic modelling does not incorporate any of the other criteria that contribute to decision-making. There is a risk that other important factors that influence reimbursement decisions may be overlooked and that a focus on cost-effectiveness may be at the expense of other relevant considerations. Depending on the jurisdiction and nature of the device there can be substantial variation in how reimbursement decisions are made.^3^ Factors influencing decisions include therapeutic value, clinical need, budget impact, organisational and ethical considerations. Clearly some of these issues cannot be addressed at an early stage of product development. However, giving them consideration at an early stage may provide an opportunity to tackle any issues that might otherwise only become apparent at a late stage of product development and hamper reimbursement and or uptake. In HTA, a technology is considered within a context (eg, taking into account the healthcare and wider social considerations) while early economic modelling is limited to cost-effectiveness and may be context-free.

### Parameter Uncertainty

Economic evaluation is a data driven discipline that seeks to synthesise evidence from a wide range of sources. Early modelling is often carried out in the absence of what might be considered high quality data. Indeed, in some cases it may be undertaken in the absence of any clinical effectiveness data, with a view to determining how effective a treatment will need to be for it to have development value. By carrying out early economic modelling when certain key parameters are unknown, uncertainty is maximised. In the study by Grutters et al, the analyses were deterministic with uncertainty accounted for through scenario analyses. While the approach is understandable as it avoids expert elicitation that may be time-consuming or challenging,^4^ it also means that probabilistic statements cannot be made. So while a scenario analysis can be constructed to show potential value, it is unclear what the likelihood of such a scenario is. This may explain why for all products there was a scenario under which they may potentially be considered cost-effective. Some aspects of uncertainty will be reduced or eliminated with further development (eg, cost of producing the technology), while others will always remain known with imprecision (eg, clinical effectiveness).^5^

### Incremental Development

The use of early economic modelling could encourage a very incremental approach to technology development. That is, it may lead to the highly strategic roll-out of product revisions. While that is the right of the manufacturer, there is an ethical question of the deferred release of innovations that have demonstrated clinical benefit. While this situation can and doubtless does arise at present, early economic modelling may result in a more systematic approach to incremental development.

### Model Quality

An estimate of cost-effectiveness is only as good as the model. Normally the risk of inaccurate model outputs informing a decision is taken on by the payer. In this case, the risk is taken on by the manufacturer. A poorly specified model may lead to unwarranted further development, or it may lead to abandoning a potentially valuable product. The manufacturer would therefore want to be confident that the model is accurate. Furthermore, there are a wide variety of methods for using early economic evaluation in decision-making and the choice of approach could influence interpretation of how to proceed with the product.^6^ It should also be noted that the device modelled could be one specific type, and the results of the analysis are then unlikely to be generalizable to other related devices. Taking a model developed for one device and applying to a similar device may give rise to inaccurate findings.

## Future Directions

### Applicability of Reported Findings

While the study by Grutters et al gives some indications of the use of early economic modelling, it leaves some unanswered questions. The study was based on consecutive technologies and could not be described as a random sample. Perhaps these technologies were put forward for early assessment precisely because they had perceived value. Given that all products were deemed to have potential value, the use of early economic modelling may have good sensitivity but poor specificity in that regard and may therefore be more useful for directing evidence gathering.^7^ The data were based on the first 32 assessments, and may capture an evolving process and learning curve that may not be representative of a steady state process. The relatively small sample size also leaves questions about the generalizability of the findings.

### Manufacturer Perspective

Much of the research on early economic evaluation has focused on methodology.^7^ What is perhaps lacking is reporting of the manufacturers’ experience of engaging in early economic modelling; we do not know how they perceived the value of the process. It would also be useful to have a follow-up analysis of what happened to the 30 technologies, to understand how early economic modelling influenced further development and to know if the realised effect size was anything like initial estimates and whether funders accept the economic evaluation findings.

As data collection and model development can be time-consuming and costly, it would be useful to know from the manufacturer’s perspective, under what conditions is the cost of carrying out early economic evaluation greater than the value of on-going product development.

### Dynamic Environment

Many of the technologies include devices that may be subject to rapid development and innovation both by the manufacturer and by competitors. The findings of an early economic model could be rendered redundant quite quickly due to the arrival of competitors, changing the landscape of relevant comparators. Of course, this issue impacts on the assessment of devices generally, so it is not limited to early assessment.

## Conclusion

While it is of questionable generalizability, the study by Grutters et al suggests that early economic modelling of devices may be beneficial. Early economic modelling can help to direct development effort by manufacturers to ensure resources are invested in viable products. Early economic modelling may have low specificity to identify low-value technologies, but have utility for directing data collection efforts to reduce uncertainty. The use of early economic modelling may turn the willingness-to-pay threshold into a target. By restricting early analysis to economic modelling, important issues that are included in a full HTA and influence reimbursement decisions may be overlooked. Future research should explore the manufacturer perspective of the utility of early economic modelling, and also to explore the accuracy of early model estimates.

## Ethical issues

Not applicable.

## Competing interests

Authors declare that they have no competing interests.

## Authors’ contributions

CT conceived and drafted the article. MR reviewed and edited the article.

## References

[1] Grutters JPC, Govers T, Nijboer J, Tummers M, van der Wilt GJ, Rovers MM (2019). Problems and promises of health technologies: the role of early health economic modeling. Int J Health Policy Manag.

[2] Ijzerman MJ, Steuten LM (2011). Early assessment of medical technologies to inform product development and market access: a review of methods and applications. Appl Health Econ Health Policy.

[3] Callea G, Armeni P, Marsilio M, Jommi C, Tarricone R (2017). The impact of HTA and procurement practices on the selection and prices of medical devices. Soc Sci Med.

[4] Cao Q, Postmus D, Hillege HL, Buskens E (2013). Probability elicitation to inform early health economic evaluations of new medical technologies: a case study in heart failure disease management. Value Health.

[5] Girling A, Young T, Brown C, Lilford R (2010). Early-stage valuation of medical devices: the role of developmental uncertainty. Value Health.

[6] Markiewicz K, van Til JA, MJ IJ (2014). Medical devices early assessment methods: systematic literature review. Int J Technol Assess Health Care.

[7] IJzerman MJ, Koffijberg H, Fenwick E, Krahn M (2017). Emerging use of early health technology assessment in medical product development: a scoping review of the literature. Pharmacoeconomics.

